# The pan-class I phosphatidyl-inositol-3 kinase inhibitor NVP-BKM120 demonstrates anti-leukemic activity in acute myeloid leukemia

**DOI:** 10.1038/srep18137

**Published:** 2015-12-17

**Authors:** Matteo Allegretti, Maria Rosaria Ricciardi, Roberto Licchetta, Simone Mirabilii, Stefania Orecchioni, Francesca Reggiani, Giovanna Talarico, Roberto Foà, Francesco Bertolini, Sergio Amadori, Maria Rosaria Torrisi, Agostino Tafuri

**Affiliations:** 1 Department of Cellular Biotechnologies and Hematology, Sapienza University of Rome, Rome, Italy; 2 Department of Clinical and Molecular Medicine, “Sant’Andrea” Hospital, Sapienza University of Rome, Rome, Italy; 3 Division of Clinical Haematology-Oncology, European Institute of Oncology, Milan, Italy; 4 Department of Hematology, Tor Vergata University Hospital, Rome, Italy

## Abstract

Aberrant activation of the PI3K/Akt/mTOR pathway is a common feature of acute myeloid leukemia (AML) patients contributing to chemoresistance, disease progression and unfavourable outcome. Therefore, inhibition of this pathway may represent a potential therapeutic approach in AML. The aim of this study was to evaluate the pre-clinical activity of NVP-BKM120 (BKM120), a selective pan-class I PI3K inhibitor, on AML cell lines and primary samples. Our results demonstrate that BKM120 abrogates the activity of the PI3K/Akt/mTOR signaling, promoting cell growth arrest and significant apoptosis in a dose- and time-dependent manner in AML cells but not in the normal counterpart. BKM120-induced cytotoxicity is associated with a profound modulation of metabolic behaviour in both cell lines and primary samples. In addition, BKM120 synergizes with the glycolitic inhibitor dichloroacetate enhancing apoptosis induction at lower doses. Finally, *in vivo* administration of BKM120 to a xenotransplant mouse model of AML significantly inhibited leukemia progression and improved the overall survival of treated mice. Taken together, our findings indicate that BKM120, alone or in combination with other drugs, has a significant anti-leukemic activity supporting its clinical development as a novel therapeutic agent in AML.

Over the last few decades, the prognosis for acute myeloid leukemia (AML) patients has shown no significant improvement and the frequency of relapse still remains very high with only 20–30% survival at 5 years^1^. Therefore, major efforts are being made to develop new therapeutic approaches targeting the aberrant signaling networks that sustain leukemia cell proliferation, survival and drug resistance^2^,^3^,^4^,^5^.

Phosphoinositide 3-kinases (PI3Ks) are a family of lipid enzymes divided into three classes (I–III) each with its own substrate specificity, structures and lipid products^6^,^7^,^8^. Class I PI3Ks are heterodimers composed of a p110 catalytic subunit (α, β, γ or δ) and a p85/p55/p50 regulatory subunit which are activated via tyrosine kinase or G protein-coupled receptors. Upon activation, cytosolic PI3K is recruited to the plasma membrane where it converts the lipid phosphatidyl-inositol bisphosphate (PIP2) to phosphatidyl-inositol trisphosphate (PIP3) inducing the colocalization of Akt and the phosphoinositide-dependent kinase 1 (PDK1) by their pleckstrin homology domain. Akt is then activated by PDK1 and mammalian target of rapamycin (mTOR) complex 2 (mTORC2) through two distinct phosphorylation on T308 and S473, respectively^9^,^10^. mTOR belongs to the PI3K-related kinase family and exists as two complexes, mTORC1 and mTORC2, which cooperate with Akt to control cell growth and proliferation, metabolism, transcription, protein translation and survival^10^,^11^. Given the pleiotropic functions, PI3Ks activity is tightly controlled and negatively regulated in normal cells by phosphatase and tensin homologue (PTEN) and SHIP-1/2, which dephosphorylates PIP3 in PIP2, inhibiting signaling transduction.

Deregulation of the PI3K/Akt/mTOR pathway is widely observed in human cancers^8^,^12^,^13^ and is approximately detected in 50–80% of the AML patients^14^,^15^,^16^,^17^. Over the last few years, constitutive activation of this axis in AML has been associated with poor prognosis and chemoresistance^14^,^16^,^18^. Moreover, it has been demonstrated that AML blasts heterogeneously expressed all class-I PI3K isoforms which are responsible for Akt activation, contributing to survival and proliferation of leukemic cells^4^. Therefore, a wide number of small molecules that target the PI3K/Akt/mTOR signaling at multiple levels, alone or in combination with chemotherapeutic drugs, has been investigated showing potential therapeutic efficacy in AML^19^,^20^,^21^.

NVP-BKM120 (cited hereafter as BKM120) is a highly selective pan-class I PI3K inhibitor that binds to wild type or mutated p110 catalytic subunits, thus preventing ATP bound to the active site of PI3K^22^,^23^,^24^. Several reports have demonstrated that BKM120 exerts anti-proliferative and cytotoxic effects on solid tumor^25^,^26^,^27^,^28^,^29^,^30^ and hematological malignancies^31^,^32^,^33^,^34^,^35^ through the selective inhibition of Akt activity. Interestingly, BKM120 at micromolar range also affects the mTOR activity by downregulating the levels of S6 protein kinase (p70^S6K^) and eIF4E-binding protein 1 (4EBP1), two well-known mTORC1 substrates^27^,^28^,^29^,^36^. Furthermore, it has been documented that BKM120 synergizes with well established chemotherapeutic drugs or other small molecules (i.e. mTOR or MEK inhibitors) to enhance apoptosis *in vitro* and *in vivo*^6^,^27^,^28^.

Here, we investigated the activity of BKM120 on human AML. Our results demonstrate that this drug inhibits cell proliferation and induces apoptosis on AML models and primary blasts by the selective inhibition of the PI3K/Akt/mTOR pathway activity, while not affecting normal cells. A profound modulation of the AML metabolic phenotype is associated with BKM120-induced cytotoxicity. Moreover, BKM120 strongly synergizes with the glycolitic modulator dichloroacetate (DCA), triggering apoptosis at lower doses in both AML cell lines and primary samples. Finally, BKM120 shows *in vivo* effectiveness prolonging the survival of an AML xenotransplant mouse model. Taken together, our findings demonstrate for the first time to our knowledge the efficacy of BKM120 as a novel therapeutic agent for AML.

## Results

### PI3K/Akt/mTOR signaling activation on AML cell lines and primary samples

**Figure 1:**
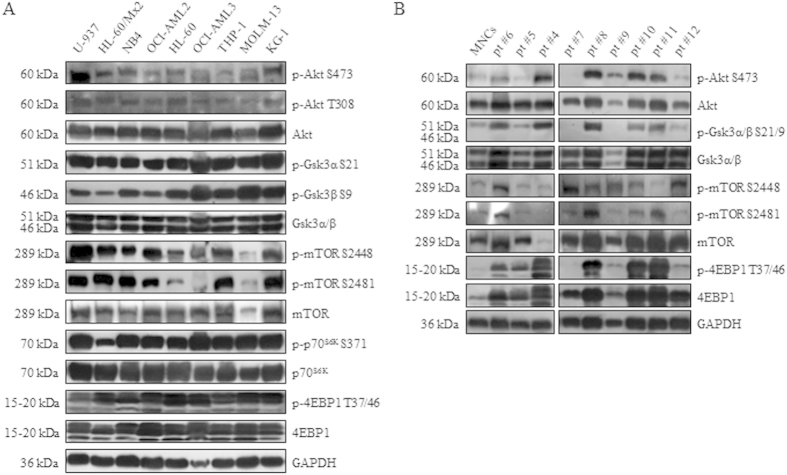
AML cell lines and primary samples show constitutively activated PI3K/Akt/mTOR pathway. Basal expression and phosphorylation levels of critical PI3K/Akt/mTOR pathway components on AML cell lines (**A**) and primary samples (**B**). Antibody to GAPDH served as loading control.

The basal expression and phosphorylation levels of critical PI3K/Akt/mTOR pathway components were first evaluated by western blot analysis on AML cell lines. Our results show that AML cell lines constitutively expressed p-Akt(S473 and T308) and p-Gsk3α/β(S21 and S9) in a heterogeneously manner, thus suggesting different levels of PI3K addiction. Moreover, a constitutive activation of the mTOR signaling was observed, as documented by the overexpression of p-mTOR(S2448 and S2481), p-p70^S6K^(S371) and p-4EBP1(T37/46), readouts of the mTORC1 and mTORC2 activities, respectively (Fig. 1A). The PI3K/Akt/mTOR activation status of primary samples included in this study was then assessed with respect to cell availability. We found that 7/9 (77.8%) of tested samples were characterized by a higher phospho/total Akt ratio (mean 2.88 ± 2.4; range 0.12–7.93) than normal MNCs (1.00) and displayed p-mTOR(S2448 and S2481) and p-4EBP1 overexpression in comparison to normal cells, thus indicating the aberrant activation of the PI3K/Akt/mTOR axis in these cases (Fig. 1B).

### BKM120 inhibits the PI3K/Akt/mTOR signaling in AML cell lines

**Figure 2:**
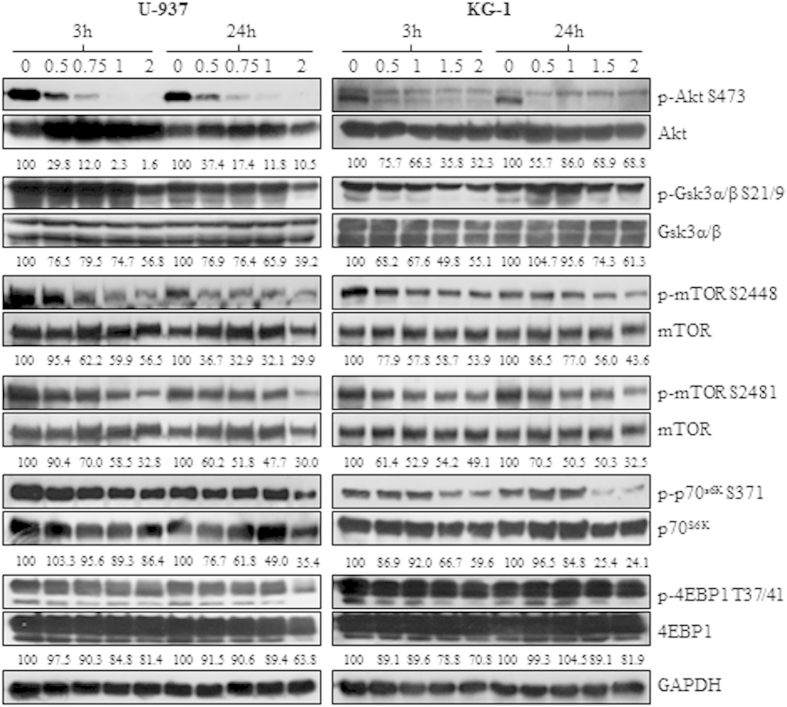
BKM120 inhibits the PI3K/Akt/mTOR signaling in AML cell lines. U-937 and KG-1 cell lines were treated up to 24 h with increasing concentration of BKM120 and protein lysates were collected at 3 and 24 h. Anti-GAPDH was used to confirm equal loading of protein samples. Results are typical of at least three separate immunoblots. Relative intensity of each protein was quantified after background subtraction by ImageJ software and the phospho/total protein ratios were expressed as percentage to that of control.

To assess whether BKM120 was able to inhibit the PI3K pathway, AML cell lines (U-937, OCI-AML2, HL-60, OCI-AML3, MOLM-13 and KG-1) were cultured for 24 h with increasing doses of BKM120 and protein lysates were obtained at 3 and 24 h. A concentration of 2 μM was not exceeded to avoid off-target effects. Densitometric analysis revealed that BKM120 induced, both at 3 and 24 h, a clear dose-dependent reduction of the phospho/total Akt ratio in all cell lines tested, although with different degree. Akt dephosphorylation was associated with the downregulation of p-Gsk3α/β(S21/9) levels, suggesting the abrogation of the PI3K downstream activity. Moreover, at the higher doses, BKM120 also decreased the phosphorylation levels of mTOR on S2448 and on S2481, thus affecting both the mTORC1 and mTORC2 activity, and led to the downregulation of p-p70^S6K^(S371) and p-4EBP1(T37/46). No changes were detected on total proteins (Fig. 2 and Supplementary Fig. S1). Thus, these findings further support the selective inhibition of the PI3K/Akt/mTOR signaling promoted by BKM120.

### BKM120 exerts anti-proliferative and pro-apoptotic effects on AML cell lines

**Figure 3:**
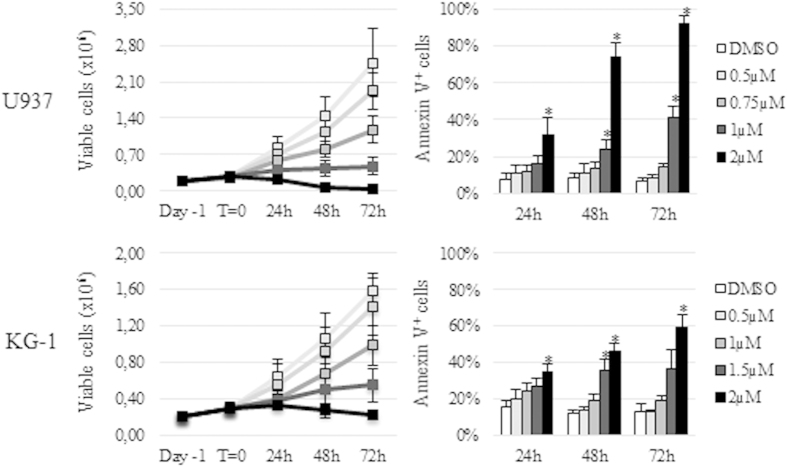
BKM120 blocks proliferation and induces apoptosis in AML cell lines. U-937 and K-G1 cell lines were plated at concentration of 0.2 × 10^6^/mL and treated with BKM120 0.5–2 μM up to 72 h. Viable cells were quantified by Trypan Blue exclusion. Apoptosis induction was determined by AnnV/PI flow cytometry. Data are represented as mean ± SD of five replicates. * for p < 0.001 compared to DMSO.

To evaluate whether PI3K inhibition resulted into cell death, AML cell lines were cultured up to 72 h with increasing concentrations of BKM120 or DMSO. Cell viability and apoptosis induction were assessed by Trypan Blue exclusion and AnnV/PI staining, respectively. BKM120 exhibited dose- and time-dependent anti-proliferative and pro-apoptotic effects on all cell lines, irrespective of their PI3K activation status, showing IC_50_s values between 0.7 and 1.2 μM (Supplementary Table S1). On the most sensitive cell line, U-937, a 24 h exposure resulted in a profound cell growth reduction and in a significant (p < 0.001) increase of AnnV/PI positive cells from 6.5% ± 0.1 (DMSO) to 91.7% ± 0.1 with 2 μM BKM120. Conversely, on the less sensitive cell line KG-1, apoptotic cells reached only 59.2% ± 0.1 (p < 0.001) after 72 h of 2 μM BKM120, still in the presence of a significant (p < 0.001) inhibition of cell growth at lower doses (Fig. 3). BKM120 cytotoxicity was preceded by a temporary G_2_/M block that was rapidly followed by induction of apoptosis, as demonstrated by the increase of the subG_0_/G_1_ peak at later times of treatment (data not shown). Together, these data confirmed the ability of BKM120 to impair cell cycle progression and mitosis regulation which ultimately turn into apoptosis.

### BKM120 affects the PI3K/Akt/mTOR activity triggering apoptosis in primary AML samples

**Figure 4:**
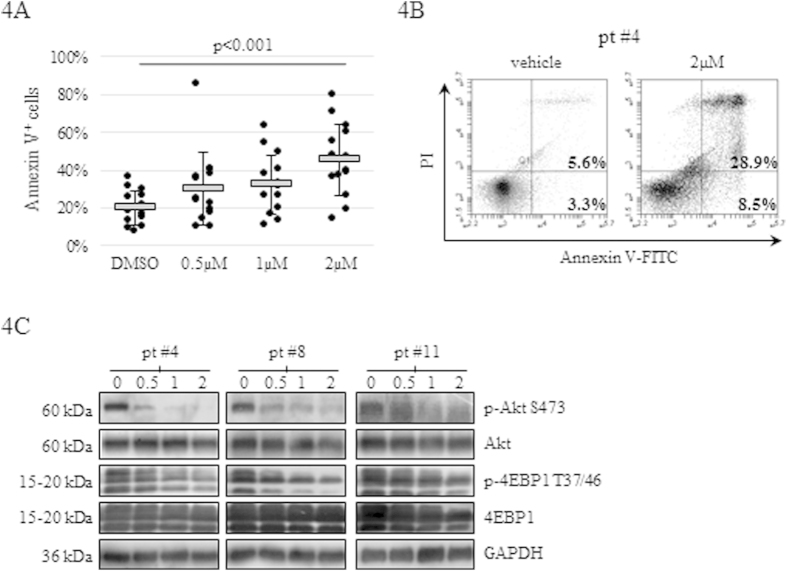
BKM120 exerts pro-apoptotic activity on primary AML cells but not affects normal MNCs. (**A**) Effects on *de novo* primary AML samples incubated with the indicated concentrations of BKM120 up to 144 h. Data from each samples are shown as individual symbols on the figure. The horizontal grey lines and error bars represent mean values and SD, respectively. (**B**) Flow cytometry analysis of BKM120-induced apoptosis on the chemo-resistant AML sample #4 after 144 h of exposure (**C**) Western blot analysis of three AML primary samples after 24 h of exposure to increasing concentrations of BKM120. Anti-GAPDH was used as loading control.

To evaluate the effectiveness of BKM120 as a potential therapeutic agent in AML, we investigated whether drug treatment could downregulate the PI3K activity and induce apoptosis on primary AML samples. AML blasts (n = 15) from BM or PB were treated up to 144 h with increasing concentration of BKM120. Cell viability and apoptosis induction were evaluated by Trypan Blue exclusion and AnnV/PI staining, respectively. Protein lysates, according to cell availability, were obtained after 24 h of exposure from 3 AML samples. BKM120 treatment decreased blast counts in all *de novo* samples analyzed, triggering at 144 h an increase of AnnV positive cells from 20.1 ± 8.9% (DMSO) to 45.4 ± 19.1% (p < 0.001) at 2 μM (Fig. 4A and Supplemental Fig. 2). Of note, BKM120 proved its efficacy also on the two chemo-resistant AML samples (#4 and #18) (Fig. 4B). Western blot analysis on 3 out 3 primary specimens confirmed that BKM120 dose-dependently inhibited the PI3K/mTOR activity also on primary blasts, as demonstrated by the downregulation of p-Akt(S473) and p-4EBP1(T37/46) (Fig. 4C). Finally, BKM120 was tested on resting and phytohaemagglutinin (PHA)-activated MNCs isolated from 5 healthy volunteers failing to show considerable induction of apoptosis (Supplementary Fig. S3).

### BKM120 modulates the metabolic phenotype of AML cells

**Figure 5:**
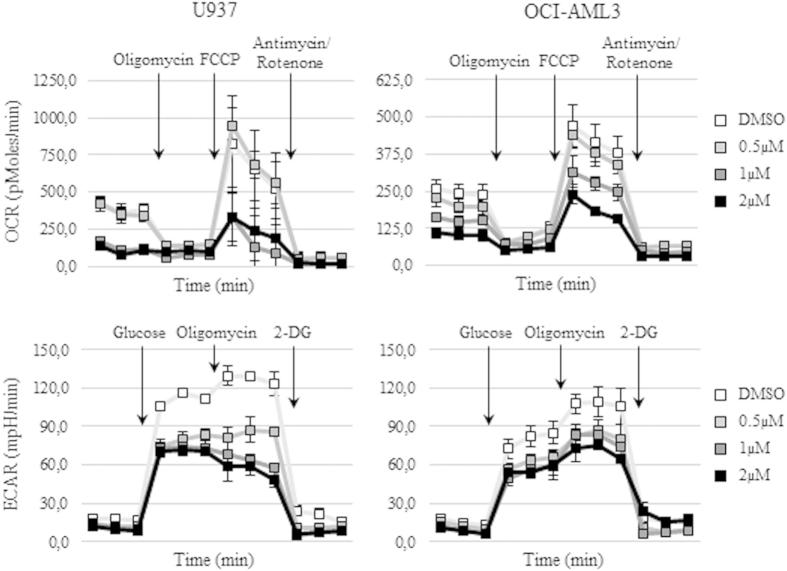
BKM120 modulates the metabolic phenotype of AML cell lines. Original Seahorse data adapted from Mito (upper panels) and GlycoStress tests (lower panels) on the indicated AML cell lines after 24 h of treatment with increasing concentrations of BKM120 or DMSO.

**Figure 6:**
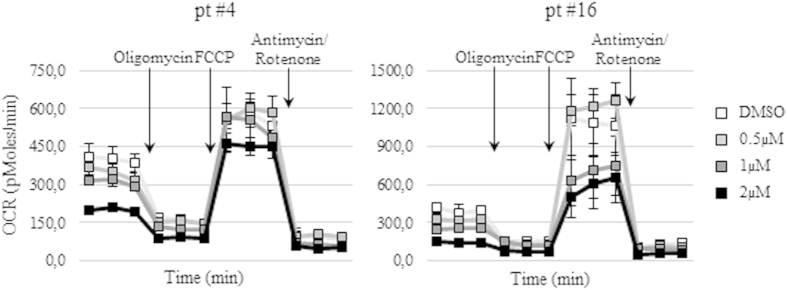
BKM120 affects the oxidative capabilities of primary AML samples. Original Seahorse data adapted from MitoStress tests on two primary AML cells after 72 h of treatment with increasing concentrations of BKM120 or DMSO.

Given the role of the PI3K/Akt/mTOR pathway in the regulation of cell metabolism, we asked whether BKM120 could modulate the metabolic phenotype of AML cell lines and primary samples. AML cell lines, U-937 and OCI-AML3 were incubated with DMSO or BKM120 (0.5–2 μM) for 24 h and the oxygen consumption rate (OCR) and the extracellular acidification rate (ECAR) were assessed by the XF24 Flux Analyzer. BKM120 exposure profoundly affected the oxidative metabolism of both AML cell lines. Indeed, on the U-937, 24 h of treatment induced a clear dose-dependent decrease of the basal and maximal respiration (72.9% and 59.9% at 2 μM) as well of the mitochondrial ATP production (97.0%) when compared to their controls. Similarly, the OCR of OCI-AML3 cell line resulted decreased in a dose-dependent manner after exposure to BKM120 (Fig. 5, upper panels). In addition, BKM120 treatment was able to reduce the basal glycolysis (30.0% and 25.8% at 2 μM) and the glycolytic capacity (53.9% and 35.9%) of both AML cell models in a dose-dependent fashion (Fig. 5, lower panels). Bioenergetic perturbations promoted by BKM120 were then evaluated following 72 h of treatment on 2 primary samples (#4 and #16) which were characterized by different clinical features (chemo-resistant vs *de novo* AML). According to cell line results, all measured rates of the oxygen consumption (Fig. 6A) and of the glycolytic status (data not shown) in primary specimens were significantly (p < 0.05) reduced upon treatment with BKM120. Hence, these results demonstrate that metabolism alterations induced by BKM120 might contribute to the cytotoxicity of this compound.

### BKM120 synergizes with DCA to enhance cytotoxicity in AML cell lines and primary samples

**Figure 7:**
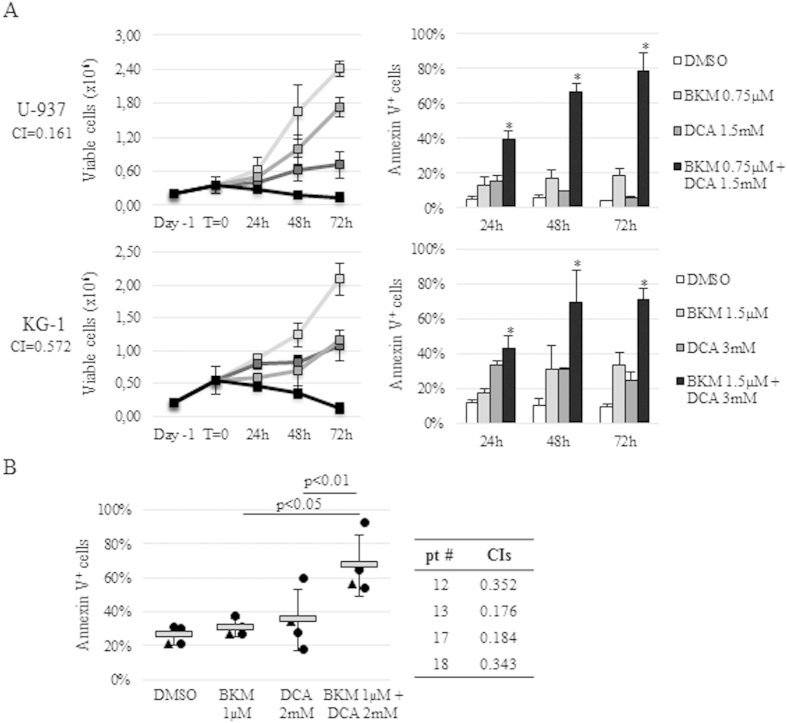
Synergistic effects of BKM120 with DCA on AML cell lines and primary samples. (**A**) Effects on U-937 and KG-1 cell lines cultured with BKM120, DCA or their combination up to 72 h. Cell growth and apoptosis were evaluated by Trypan Blue exclusion and AnnV/PI staining. Data are representative of one of multiple doses tested and are expressed as mean ± SD of three independent replicates. CI values are indicated for each cell line. (**B**) Effects on primary AML samples exposed to BKM120, DCA or their combination for 144 h. Data from *de novo* (circles) and chemo-resistant (triangle) samples are shown as individual symbols on the figure and represent one of multiple concentrations tested. The horizontal grey lines and error bars represent mean values and SD, respectively. CI values are reported for each patient. * for p < 0.05 compared to single drug treatments.

According to the metabolic importance of the PI3K pathway, we explored a novel therapeutic strategy combining BKM120 with DCA at doses around or below their IC_50_s on AML cell models and primary specimens. The U-937, HL-60 and KG-1 cell lines were exposed to increasing concentrations of BKM120 (0.5–1.5 μM) and DCA (1–3 mM) alone and in combination at a fixed ratio (1:2000) and cytotoxicity was monitored by FACS analysis up to 72 h. As shown in Fig. 7A, the combination of BKM120 and DCA was more effective in inhibiting cell growth and promoting massive apoptosis induction on all cell lines tested whereas single drugs did not. CIs were below 1 (range 0.2–0.6), thus indicating the strong synergistic interaction between these two drugs. Combined treatment was strikingly effective also on the DCA-resistant cell line, HL-60, increasing the percentage of apoptotic cells at 72 h from 17.6 ± 10.6 (BKM120 1.5 μM) and 14.3 ± 3.1 (DCA 3 mM) to 97.5 ± 0.1 (combination) (Supplementary Fig. S4A). BKM120/DCA combination was then assessed on 4 primary AML specimens, 3 *de novo* (#12, #13 and #17) and 1 chemo-resistant sample (#18), confirming its efficacy to decrease blast counts and induce apoptosis in a more pronounced way than single agents (Fig. 7B and Supplementary Fig. S4B).

### BKM120 impairs AML tumor growth and prolongs survival *in vivo*

**Figure 8:**
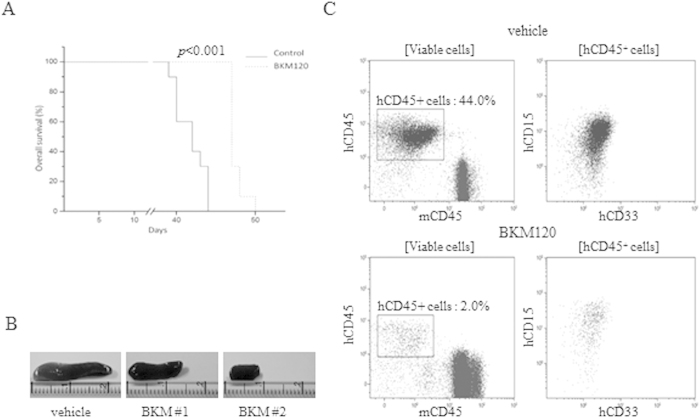
BKM120 reduces AML tumor growth and human cell engraftment, prolonging the survival of treated-mice. NSG mice (n = 28) were intravenously injected with 1 × 10^6^ HL-60 cells. One day after cell transfer, mice were randomized into two groups to receive 40 mg/kg of BKM120 (treated group) or vehicle (control group). (**A**) Survival curves of control and treated groups. (**B**) Representative spleen size of vehicle vs BKM120-treated mice. (**C**) Tumor engraftment was evaluated at day 45.

Cytotoxic efficacy of BKM120 was finally investigated in a xenotransplant mouse model of AML. NSG mice (n = 28) were intravenously injected with 1 × 10^6^ HL-60 cells and, one day after cell transfer, were randomized into two groups to receive 40 mg/kg of BKM120 or vehicle. BKM120 treatment markedly inhibited leukemia progression inducing a significant (p < 0.001) improvement of overall survival when compared to the control mice (Fig. 8A). Treatment was well tolerated as suggested by the maintenance of body weight and the lack of other signs of toxicity (data not shown). Thirty days after cell injection, AML-related symptoms were observed in the vehicle group (animals were hunchbacked and ruffled coat) and mice began to die. At necropsy, mice had enlarged spleen in comparison to BKM120-treated group (Fig. 8B). At day 45 after cell transfer, flow cytometry evaluation of the circulating human CD45^+^ cells in the murine PB was perfomed to assess AML engraftment showing that BKM120-treated mice carried significantly lower leukemia burden in the PB in comparison to the vehicle-treated group (Fig. 8C).

## Discussion

It is well established that an aberrant expression of the PI3K/Akt/mTOR pathway plays a pivotal role in cancer cell proliferation, survival and chemotherapy resistance through the alteration of physiologic cell cycle progression, differentiation and growth^13^,^19^,^37^. Over the last few years, several studies have shown the constitutive activation of this axis in different hematologic malignancies, including AML, providing the rationale for new targeted approaches. Furthermore, different groups have demonstrated that the PI3K/Akt/mTOR activation is an indicator of poor prognosis and chemoresistance in AML^16^,^18^,^38^.

In the present study, we investigated for the first time the efficacy of the pan-class I PI3K inhibitor BKM120 as a potential therapeutic inhibitor of the PI3K/Akt/mTOR signaling network in AML. Previous reports in solid tumors^25^,^26^,^27^,^28^,^29^,^30^ and hematologic malignancies^31^,^32^,^33^,^34^,^35^ have shown that BKM120, alone or in combination with other drugs (e.g. standard chemotherapy or other small molecules), inhibits cell cycle progression and promotes apoptosis induction through the selective inhibition of the PI3K/Akt/mTOR pathway activity, thus supporting its potential clinical role in cancer. In the present study, we examined the *in vitro* cytotoxic potential of BKM120 on a wide panel of human AML cell lines characterized by different PI3K/Akt/mTOR pathway activation patterns. Our results demonstrate that BKM120 significantly reduced cell viability and triggered marked apoptosis induction in a dose- and time-dependent manner in all cell lines tested, regardless of their PI3K dependence. Accordingly to previous reports^24^,^29^,^35^,^36^, BKM120-induced cytotoxicity in AML cell models was preceded by a temporary increase of cells in the G2/M phase of cell cycle, further supporting that BKM120 impairs cell cycle progression and mitosis regulation which ultimately turn into apoptosis. Clinical relevance of BKM120 was further confirmed on primary AML samples obtained from *de novo* and chemo-resistant patients with different PI3K/Akt/mTOR pathway activation and on MNCs obtained from healthy volunteers. BKM120 exposure was able to decrease the viability of leukemic blasts in a dose-dependent manner in all AML samples tested, *de novo* and chemo-resistant, resulting in a significant induction of apoptosis. The median IC_50_ was 1.52 μM, significantly lower than the maximum plasma concentration of BKM120 (4 μM) obtained in a phase I clinical trial after administration of the maximum tolerated dose of this drug^39^. By contrast, no considerable cytotoxicity was observed on resting and activated normal MNCs, thus suggesting the existence of a therapeutic window in humans. This evidence is further supported by the lack of relevant toxicity in patients with advanced leukemias treated with BKM120 in a phase I/II clinical trial^40^. The efficacy of BKM120 in AML cells was strongly associated with the abrogation of the PI3K/Akt/mTOR signaling activity. As indicated by the densitometric analysis, BKM120 exposure dose-dependently reduced the phospho/total protein ratios of critical PI3K/Akt/mTOR pathway components, emphasizing once again the selective target inhibition prompted by this drug on cancer cells. We also documented a downregulation of mTORC1/2 phosphorylation levels as well as of the mTORC1 downstream targets, p70^S6K^ and 4EBP1. These data are in agreement with previous results^27^,^28^,^29^,^36^ showing the capacity of BKM120 at higher doses to inhibit also the catalytic activity of the mTOR kinase. Importantly, target inhibition was demonstrated on primary samples and was associated with a clear dephosphorylation of 4EBP1, a translational repressor involved in the synthesis of several oncogenic proteins^41^,^42^.

Recently, it has been established that the PI3K/Akt/mTOR pathway strongly influences cellular metabolism through the direct regulation of Akt on metabolic enzymes and, indirectly, through the stimulation of mTORC1 activity^43^,^44^,^45^. Moreover, the Akt activation status has been shown to mediate the metabolic effects in human leukemic cells^46^. Therefore, we investigated whether BKM120 exposure could modulate the metabolic phenotype of AML cell lines and primary samples. In agreement with a recently published work on hepatocellular carcinoma^27^ we found that inhibition of the PI3K/Akt/mTOR signaling by BKM120 significantly reduced the basal and maximal respiration as well as the percentage of ATP synthesis from mitochondria in a dose-dependent manner when compared to their controls. Moreover, BKM120 exposure affects the glycolytic status of AML cells, decreasing both the basal glycolysis and the glycolytic capacity. These effects were clearly apparent on all tested AML cells, including primary samples, thus confirming that BKM120-induced modulation of altered metabolic fluxes in leukemic cells result into the induction of apoptosis.

Leukemic cells, like other cancer cells, are characterized by a reprogrammed glucose metabolism which provides energy and biosynthetic precursors or metabolic intermediates to sustain tumor needs^47^,^48^,^49^. Altered glucose metabolism in AML has been shown to be closely associated with therapeutic resistance and clinical outcome^46^,^47^, strongly contributing to the decreased sensitivity to some anti-leukemic agents such as arabinofuranosyl cytidine (Ara-C). Conversely, inhibition of glycolysis suppressed AML cell proliferation and potentiated drug-induced cytotoxicity^47^. Of note, Scotland *et al.*^46^ showed a striking correlation between PI3K/Akt/mTOR constitutive activation and susceptibility to glycolitic inhibitor. We thus decided to explore a novel therapeutic strategy combining DCA, a well-known metabolic modulator previously tested by us and other groups^47^,^50^ also on AML cell lines, with BKM120 at doses around or below their IC_50_s. According to other reports showing the efficacy of simultaneous inhibition of Akt and glycolysis^51^, we could demonstrate that BKM120/DCA combination synergistically inhibits cell lines proliferation and promotes apoptosis induction at significantly lower doses than single drugs. Importantly, the efficacy of the combination was also confirmed on both *de novo* and chemo-resistant primary samples, emphasizing the clinical relevance of targeted approaches simultaneously inhibiting altered signalling and the glycolitic pathway in AML cells. Finally, the evaluation of the *in vivo* potential of BKM120 on xenotransplant AML mouse models demonstrated that this compound efficiently inhibits cell engraftment, delays tumor development and prolongs the survival of AML-bearing mice without inducing any side effects or weight loss.

In conclusion, our pre-clinical results have documented that BKM120, as single agent or in combination with other drugs (i.e. glycolytic modulators), has a significant anti-leukemic activity towards AML cell lines and primary samples, thus supporting its clinical evaluation as a therapeutic agent for the management of AML.

## Methods

### Materials

BKM120, kindly provided by Novartis (Basel, Switzerland), was dissolved in dimethylsulphoxide (DMSO) to a stock concentration of 10 mM for *in vitro* studies or prepared as described below for *in vivo* administration. DCA, a pyruvate dehydrogenase kinase inhibitor, was purchased from Sigma-Aldrich (St Louis, MO, USA) and freshly diluted in Dulbecco’s PBS just before each experiment.

 For western blot analysis, the anti-GAPDH was obtained from Millipore Corporation (Billerica, MA, USA) while all other primary antibodies were bought from Cell Signaling Technology (Danvers, MA, USA). Secondary HRP-conjugated antibodies and x-ray films were purchased from GE Healthcare (Piscataway, NJ, USA). Electrochemiluminescence (ECL) reagent and non-fat dry milk were from Euroclone (Milan, Italy).

### Cell culture and primary samples

The U-937, HL-60, HL-60Mx2, THP-1 and KG-1 cell lines were purchased from ATCC catalogue while the NB4, OCI-AML2, OCI-AML3 and MOLM13 were from DSMZ cell bank. All AML cell lines were grown at a concentration of 0.2 × 10^6^/mL in RPMI 1640 medium (Euroclone) supplemented with 10% fetal bovine serum, 2 mM l-glutamine and 1% penicillin-streptomycin and maintained at 37 °C and 5% CO_2_. Primary samples from AML patients (n = 18) and mononuclear cells (MNCs) from healthy donors (n = 5) were collected after written informed consent from all subjects, purified by Lympholyte-H (Cederlane, Ontario, Canada) density-gradient centrifugation and plated at a concentration of 1 × 10^6^/mL under the same culture conditions of cell lines. Experimental protocols were in accordance to the Helsinki Declaration and were approved by the Sapienza Institutional Review Board (Prot. #158/10 signed in February 18^th^, 2010). The characteristics of patients are reported in Supplementary Table S2.

### Apoptosis and cell cycle analysis

MTT [3–4,5-dimethylthythiazol-2-yl)-2,5-diphenyltetrazolium bromide] assays (Sigma-Aldrich) were performed both to establish the optimal concentration range of drug treatment and to evaluate growth inhibitory effects on AML cell lines. Briefly, cells were seeded into 24-well plates and incubated for 72 h with increasing concentrations of BKM120 or with DMSO following which 100 μL of each condition were transferred in triplicate into a 96-well plate. Then, 10 μL of MTT were added to each well and plates were incubated for 3 h at 37 °C and 5% CO_2_. After 3 h, MTT was dissolved with 100 μL of solubilization solution and absorbance was measured at 570 nM with a LT-4000 spectrophotometer (Euroclone). All experiments were performed at least 3 times and results were expressed as mean ± standard deviation (SD).

Apoptosis induction was examined up to 72 h for AML cell lines and up to 144 h for primary AML samples using Annexin V-fluorescein isothiocyanate (FITC)/propidium iodide (PI) staining, as previously described^52^. At the same time, cell cycle analyses were performed by acridine-orange (AO) technique, according to Tafuri *et al.*^53^. Flow cytometry data were collected by FACSCan and Accuri C6 flow cytometers (BD Biosciences, CA, USA) and analyzed with the appropriate software.

### Cell signaling and metabolic analysis

Cell lines and primary samples exposed to BKM120 were lysed in ice-solution containing 10 mM NaF, 1 mM Na_3_VO_4_, 150 mM NaCl, 1 mM MgCl_2_, 1 mM CaCl_2_, 0.1% NaN_3_, 10 mM iodoacetamide, 3 mM PMSF and 1% Triton X-100 (Sigma-Aldrich) supplemented with protease inhibitor cocktail (Roche Diagnostic Corp, Indianapolis, IN, USA)^54^. Equal amounts of proteins were subjected to SDS–PAGE. After electrotransfer on nitrocellulose (Bio-Rad Laboratories, CA, USA), membranes were blocked in 5% non-fat dry milk and incubated overnight with primary antibodies, according to manufacturer’s instruction. Blots were then probed with HRP-conjugated secondary antibodies for 1 h at room temperature and developed using ECL reagent. Resulting signals were collected on x-ray films, digitally scanned and quantified using Image J software (NIH, Bethesda, MD, USA)^54^.

Bioenergetic changes induced by drug treatment were evaluated using the XF24 Flux analyzer (Seahorse Biosciences, Billerica, MA, USA) which performs real-time measurements of OCR and ECAR directly on intact cells. Briefly, cell lines and primary samples were harvested from their cultures after 24 h and 72 h of treatment, respectively, washed twice in XF Base medium (Seahorse Biosciences) supplemented with 2 mM l-glutamine, 11 mM glucose and 1,2 mM pyruvate for MitoStress tests or with 2 mM l-glutamine for GlycoStress tests, and seeded in a 24-well XF test plate previously treated with 50 μgr/mL poly-lysine (Sigma-Aldrich) at a density of 0.5 × 10^6^/well and 1.5 × 10^6^/well for cell lines and primary blasts, respectively. Each condition was plated at least in quadruplicate to minimize handling variations. XF Stress kits were purchased from Seahorse Biosciences and performed according to manufacturer’s instructions.

### Mice and human leukemia transplants

Experiments were carried out on non-obese diabetic severe combined immunodeficient (NOD/SCID) interleukin-2 receptor g (IL-2Rg)–null (NSG) mice, 6 to 8-weeks-old. NSG mice were bred and housed under pathogen-free conditions in the animal facilities at the European Institute of Oncology–Italian Foundation for Cancer Research Institute of Molecular Oncology (IEO-IFOM, Milan, Italy). All animal experiments were carried out in strict accordance with the Italian laws (DLvo 26/2014 and following additions) and were approved by the institutional committee. For induction of acute leukemia, 1 × 10^6^ HL-60 cells were injected intravenously through the lateral tail vein in non-irradiated mice. Human engraftment was defined by means of percentage of human cells in peripheral blood. BKM120 was prepared fresh daily just before gavaging at concentration of 40 mg/kg, dissolved in 10% N-methyl-2-pyrrolidone and 90% PEG 300. One day after HL-60 cells injection, mice were randomized in two groups: first group was treated daily for 40 days with BKM120 while the other was administered with vehicle as control.

### *In vivo* flow cytometry analysis

Flow cytometry analyses were performed 45 days after transplant. Human cell engraftment in the peripheral blood was investigated after mechanical dissociation of the organs. The phenotype of human cells in NSG mice was evaluated using the following anti-human antibodies: anti-CD15-FITC (80H5), -CD13-PeCy7 (IMMU 103.44), -CD45-APC (J.33), -CD33-APC-Cy7 (D3HL60.251) from Beckman-Coulter (Irving, TX, USA) and anti-mouse CD45-PE (30-F11) from BD Biosciences to exclude murine cells contamination. Cell suspensions were evaluated by a FACSCalibur (BD Biosciences) using analysis gates designed to exclude dead cells, platelets and debris. Percentages of stained cells were determined and compared to appropriate negative controls. Seven-aminoactinomycin D (7AAD) from Sigma-Aldrich was used to enumerate viable, apoptotic and dead cells.

### Data analysis and statistics

For all *in vitro* results, at least three independent experiments were performed to determine mean and SD values. IC_50_s were calculated using the CalcuSyn software (Biosoft, Cambridge, MA) based on the number of live cells at 72 h. For combination experiments, synergism, additive effects, and antagonism were assessed using the Chou–Talalay method and the CalcuSyn software. CI values of 1 were considered as additive, <1 as synergistic and >1 antagonistic.

For *in vivo* study, the sample size has been established by the calculator for sample size requirement for log rank test in survival analysis (https://www.statstodo.com/SSizSurvival_Pgm.php) setting the probability of type I error (α) at 0.05, the power (1-β) at 0.80, and the minimum difference to be found significant at 60%. The size required to highlight such a difference has been found at least at 14 animals per cohort. Statistical comparisons were performed using the two-sided paired Student’s *t* test, analysis of variance (ANOVA) and linear regression when data were normally distributed or the non-parametric analyses of Spearman and Mann-Whitney when data were not normally distributed. Values of *p* < 0.05 were considered as statistically significant.

## Additional Information

**How to cite this article**: Allegretti, M. *et al.* The pan-class I phosphatidyl-inositol-3 kinase inhibitor NVP-BKM120 demonstrates anti-leukemic activity in acute myeloid leukemia. *Sci. Rep.* **5**, 18137; doi: 10.1038/srep18137 (2015).

## Supplementary Material

Supplementary Information
