# Erratum: The pan-class I phosphatidyl-inositol-3 kinase inhibitor NVP-BKM120 demonstrates anti-leukemic activity in acute myeloid leukemia

**DOI:** 10.1038/srep20743

**Published:** 2016-02-05

**Authors:** Matteo Allegretti, Maria Rosaria Ricciardi, Roberto Licchetta, Simone Mirabilii, Stefania Orecchioni, Francesca Reggiani, Giovanna Talarico, Roberto Foà, Francesco Bertolini, Sergio Amadori, Maria Rosaria Torrisi, Agostino Tafuri

Scientific Reports 5: Article number: 1813710.1038/srep18137; published online: 12172015; updated: 02052016

This Article contains errors in the Author Contributions Statement.

“M.A. designed and performed the research, analyzed data and wrote the manuscript; M.R.R. designed the research, analyzed data and edited the manuscript; S.M. performed metabolic experiments R.L. performed functional assays; S.O., F.R. and G.T. performed *in vivo* experiments; R.F. provided clinical samples; F.B. designed *in vivo* research and edited the manuscript; S.A. conceived of the research, provided clinical samples and edited the manuscript; A.T. conceived the research and edited the manuscript. All authors reviewed and approved the manuscript.”

should read:

“M.A. designed and performed the research, analyzed data and wrote the manuscript; M.R.R. designed the research, analyzed the data and edited the manuscript; R.L. performed functional assay; S.M. performed metabolic experiments; S.O., F.R. and G.T. performed *in vivo* experiments; R.F. provided clinical samples; F.B. designed *in vivo* research and edited the manuscript; S.A. conceived the research, provided clinical samples and edited the manuscript; M.R.T. edited the manuscript; A.T. conceived the research and edited the manuscript. All authors reviewed and approved the manuscript.”

