# Supplementary material for: The pan-class I phosphatidyl-inositol-3 kinase inhibitor NVP-BKM120 demonstrates anti-leukemic activity in acute myeloid leukemia
Source: Sci Rep. 2015 Dec 17;5:18137. doi: 10.1038/srep18137 (PMC4682184; doi:10.1038/srep18137)
Supplement: Supplementary Information [file srep18137-s1.doc]

**The pan-class I phosphatidyl-inositol-3 kinase inhibitor NVP-BKM120 demonstrates anti-leukemic activity in acute myeloid leukemia.**

*Matteo Allegretti^1+^, Maria Rosaria Ricciardi^2+^, Roberto Licchetta^1^, Simone Mirabilii^1^, Stefania Orecchioni^3^, Francesca Reggiani^3^, Giovanna Talarico^3^, Roberto Foà^1^, Francesco Bertolini^3^, Sergio Amadori^4^, Maria Rosaria Torrisi^2^ and Agostino Tafuri^2*^.*

1 Department of Cellular Biotechnologies and Hematology, Sapienza University of Rome, Rome, Italy

2 Department of Clinical and Molecular Medicine, Hematology, "Sant'Andrea" Hospital, Sapienza University of Rome, Rome, Italy

3 Division of Clinical Haematology-Oncology, European Institute of Oncology, Milan, Italy

4 Department of Hematology, Tor Vergata University Hospital, Rome, Italy

^+^These authors contributed equally to this work.

* Corresponding author:

e-mail: [agostino.tafuri@uniroma1.it](mailto:agostino.tafuri@uniroma1.it)

**Supplementary Tables**

| **Cell line** | **IC_50_ (µM)** |
| --- | --- |
| U-937 | 0.73 |
| HL-60/Mx2 | 0.74 |
| NB4 | 0.77 |
| OCI-AML2 | 0.81 |
| HL-60 | 0.88 |
| OCI-AML3 | 1.02 |
| THP-1 | 1.05 |
| MOLM-13 | 1.13 |
| KG-1 | 1.18 |

**Supplementary Table S1. IC_50_ values determined at 72h from growth curves by CalcuSyn software.**

| **pt #** | **Age**  **(years)** | **Sex** | **Diagnosis** | **Sample type** | **WBC**  **(10^3^/μL)** | **Blasts**  **(%)** | **FAB**  **phenotype** |
| --- | --- | --- | --- | --- | --- | --- | --- |
| 1 | 49.9 | F | de novo | BM | 155.0 | 95 | M5 |
| 2 | 57.8 | F | de novo | BM | 35.3 | 95 | M1 |
| 3 | 43.7 | M | de novo | BM | 3.4 | 70 | M4 |
| 4 | 55.6 | F | chemo-resistant | PB | 87.0 | 85 | n.d. |
| 5 | 78.5 | M | de novo | PB | 23.7 | 90 | M1/M2 |
| 6 | 48.0 | M | de novo | BM | 138.0 | 85 | M5 |
| 7 | 41.3 | F | de novo | BM | 127.0 | 100 | n.d. |
| 8 | 51.4 | M | de novo | BM | 37.4 | 98 | n.d. |
| 9 | 47.8 | M | de novo | BM | 5.1 | 73 | n.d. |
| 10 | 53.7 | F | de novo | BM | 9.0 | 80 | M1 |
| 11 | 42.6 | F | de novo | BM | 204.2 | 93 | M2 |
| 12 | 34.6 | M | de novo | BM | 65.0 | 80 | M4 |
| 13 | 54.2 | M | de novo | BM | 259.1 | 70 | M4 |
| 14 | 24.7 | F | de novo | BM | 321.4 | 82 | n.d. |
| 15 | 47.0 | F | de novo | BM | 84.1 | 77 | n.d. |
| 16 | 30.8 | M | de novo | BM | 40.0 | 99 | M2 |
| 17 | 52.5 | F | de novo | BM | 35.3 | 70 | M5 |
| 18 | 79.1 | M | chemo-resistant | PB | 54.0 | 90 | M1/M2 |

**Supplementary Table S2. Clinical features of primary AML samples included in this study.** Abbreviations: n.d., not determined

**Supplementary Figures**


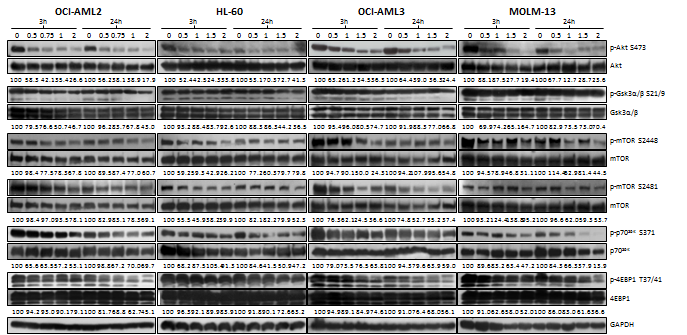


**Supplementary Fig S1. BKM120 inhibits the PI3K/Akt/mTOR signaling in AML cell lines.** AML cell models were treated with the indicated concentration of BKM120 and protein lysates were collected at 3 and 24h. Anti-GAPDH was used as loading control. Results are typical of at least three separate immunoblots. Relative intensity of each protein was quantified after background subtraction by ImageJ software and the phospho/total protein ratios were expressed as percentage to that of control.


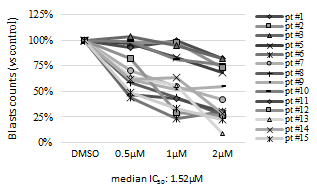


**Supplementary Fig S2. BKM120 decreases blast counts on primary AML cells.** Effects on *de novo* primary AML samples incubated with the indicated concentrations of BKM120 up to 144h. The median IC_50_, determined by CalcuSyn software after 144h of exposure, is reported.


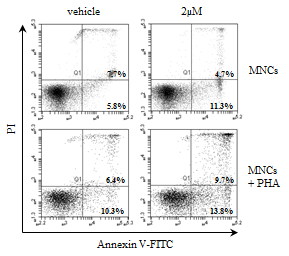


**Supplementary Fig S3. BKM120 does not affects normal and PHA-activated MNCs.** Representative flow cytometry analysis of normal and PHA-activated MNCs incubated with vehicle (DMSO) or BKM120 2µM for 24h.


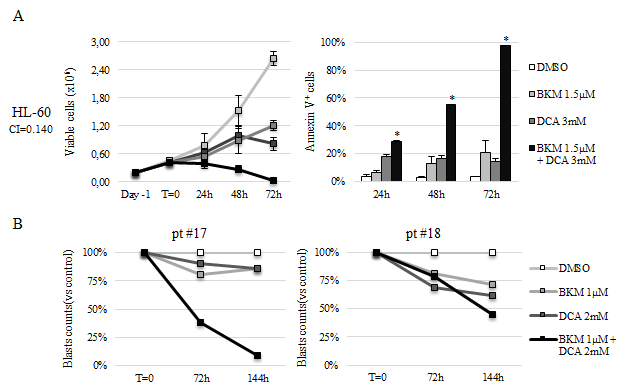


**Supplementary Fig S4. Synergistic effects of BKM120 plus DCA on HL-60 cell line and primary AML samples.** (A) Effects on the DCA-resistant HL-60 cell line cultured in the presence of BKM120, DCA or their combination up to 72h. Cell growth and apoptosis were evaluated by Trypan Blue exclusion and AnnV/PI staining. Data are expressed as mean±SD of three independent replicates. CI value, determined by Calcusyn software after 72h of exposure, is reported. (B) Effects of BKM120/DCA combination on blast counts of two representative *de novo* (pt#17) and chemo-resistant (pt#18) primary AML samples. * for p<0.05 compared to single drug treatments.
